# A novel Lnc408 maintains breast cancer stem cell stemness by recruiting SP3 to suppress CBY1 transcription and increasing nuclear β-catenin levels

**DOI:** 10.1038/s41419-021-03708-6

**Published:** 2021-05-01

**Authors:** Siyang Wen, Yilu Qin, Rui Wang, Liping Yang, Huan Zeng, Pengpeng Zhu, Qiao Li, Yuxiang Qiu, Shanchun Chen, Yongcan Liu, Yixuan Hou, Xi Tang, Manran Liu, Gang Tu

**Affiliations:** 1 Key Laboratory of Laboratory Medical Diagnostics, Chinese Ministry of Education, Chongqing Medical University, 400016 Chongqing, China; 2 Experimental Teaching Center of Basic Medicine Science, Chongqing Medical University, 400016 Chongqing, China; 3 Department of Laboratory Medicine, The First Affiliated Hospital of Chongqing Medical University, 400016 Chongqing, China; 4 Department of Endocrine and Breast Surgery, The First Affiliated Hospital of Chongqing Medical University, 400016 Chongqing, China

**Keywords:** Cancer stem cells, Stem cells

## Abstract

Tumor initiation, development, and relapse may be closely associated with cancer stem cells (CSCs). The complicated mechanisms underlying the maintenance of CSCs are keeping in illustration. Long noncoding RNAs (lncRNAs), due to their multifunction in various biological processes, have been indicated to play a crucial role in CSC renewal and stemness maintenance. Using lncRNA array, we identified a novel lncRNA (named lnc408) in epithelial–mesenchymal transition-related breast CSCs (BCSCs). The lnc408 is high expressed in BCSCs in vitro and in vivo. The enhanced lnc408 is critical to BCSC characteristics and tumorigenesis. Lnc408 can recruit transcript factor SP3 to CBY1 promoter to serve as an inhibitor in CBY1 transcription in BCSCs. The high expressed CBY1 in non-BCSC interacts with 14-3-3 and β-catenin to form a ternary complex, which leads a translocation of the ternary complex into cytoplasm from nucleus and degradation of β-catenin in phosphorylation-dependent pattern. The lnc408-mediated decrease of CBY1 in BCSCs impairs the formation of 14-3-3/β-catenin/CBY1 complex, and keeps β-catenin in nucleus to promote CSC-associated CD44, SOX2, Nanog, Klf4, and c-Myc expressions and contributes to mammosphere formation; however, restoration of CBY1 expression in tumor cells reduces BCSC and its enrichment, thus lnc408 plays an essential role in maintenance of BCSC stemness. In shortly, these findings highlight that the novel lnc408 functions as an oncogenic factor by recruiting SP3 to inhibit CBY1 expression and β-catenin accumulation in nucleus to maintain stemness properties of BCSCs. Lnc408–CBY1–β-catenin signaling axis might serve as a new diagnostic and therapeutic target for breast cancer.

## Introduction

Breast cancer is the most common cancer among women worldwide. Despite advances in diagnosis and treatment, the resistance to breast cancer therapies is still a challenge^1–3^. Metastasis and recurrence are the major causes of cancer-related death^4,5^, which hampers satisfactory of treatment. Therefore, more comprehensive investigations should be carried out on the mechanism of metastasis and relapse in breast cancer, which is crucial for improving the prognosis of patients.

A leading contributing factor to metastasis and treatment resistance is the heterogeneity and plasticity of the cells within tumors^6^. As a small pool of tumor cells, cancer stem cells (CSCs), which contribute to tumor heterogeneity, are characterized by self-renewal and therapy resistance, and promote initiation or tumorigenesis^7–9^. Increasing evidence has shown that many cancers, including breast cancer exist CSCs. Breast cancer stem cells (BCSCs) own unique surface markers, such as CD44^+^CD24^−/low^ ^10,11^. Recent years, BCSCs receive more and more attention. Wnt/β-catenin, hedgehog, and Notch/YAP signaling have been reported to maintain self-renewal of BCSCs^12–14^. Thus, clarifying the precise mechanism of BCSCs self-renewal and identifying novel targets for effective intervention of breast cancer is urgently needed.

LncRNAs are a sort of small nuleic acid molecule with a length of >200 nucleotides with no or little protein-coding capacity^15^. LncRNAs have been indicated to participate in various physiological and pathological processes. To date, emerging evidence have shown that lncRNAs play a pivotal role in maintaining stemness of CSCs through different mechanism^16^. For example, in gastric cancer, the enhanced ASB16-AS1 strengthens TRIM37 expression to activate NF-κB signaling and promotes stemness maintenance^17^. In MLL leukemia, LAMP5-AS1 facilitates the methyltransferase activity of DOT1L to drive leukemia cell stemness^18^. In bladder cancer, lncRNA SOX2OT sponges with miR-200c to modulate stemness phenotype^19^. In hepatocellular carcinoma (HCC), HAND2-AS1 promotes the self-renewal of liver CSCs and drives liver oncogenesis through BMP signaling^20^. Nevertheless, finding reports about the involvement of lncRNAs in BCSCs regulation at transcriptional level are rare. Herein, we identified a novel lncRNA-408, which is specifically expressed in BCSCs and plays a pivotal role in maintaining the stemness of BCSCs.

In this study, we found lnc408 was highly expressed in BCSCs. lnc408 recruited SP3 to suppress the expression of CBY1, the decreased CBY1 hampered the formation of ternary complex of 14-3-3/β-catenin/CBY1, which led to the accumulation of β-catenin in the nucleus of BCSC, and thus activated downstream signaling pathways and promoted the enrichment and maintenance of BCSCs. The lnc408/CBY1 axis might provide new insights into the mechanism of BCSC’s self-renewal and theoretical support for finding new targets of breast cancer diagnosis and therapy.

## Materials and methods

### Antibodies and reagents

The antibodies specifically against SOX2, c-MYC, 14-3-3, and β-Catenin were purchased from Abcam (UK); anti-CD44 and anti-KLF4 were products of Proteintech (USA); anti-β-actin, HRP-conjugated anti-mouse or anti-rabbit IgG were purchased from ZSGBBIO (CHN); anti-CBY1 was purchased from Santa Cruz (USA); anti-SP3 was purchased from Sigma-Aldrich (USA); anti-Nanog, anti-phosphorylated-β-catenin, anti-GAPDH and anti-PCNA were obtained from Bioworld (USA); and FITC-labeled goat anti-rabbit IgG was from Sigma-Aldrich (USA). BSA was a product of Sigma-Aldrich (USA); bFGF was obtained from Proteintech (USA); EGF and B27 supplement were from Invitrogen (Thermo Fisher, USA), and ultra-low attachment plates were purchased from Corning Corp. (USA).

### Cell culture and mammosphere formation assay

Human breast cancer cell lines BT549 or Hs578T were obtained from the American Type Culture Collection. Cells were cultured in RPMI-1640 medium supplemented with 10% FBS, 1% penicillin, and 1% streptomycin.

For mammosphere culture, 2 × 10^4^ breast cancer cells were seeded into six-well ultra-low attachment plates supplemented with sphere formation medium (DMEM/F12 containing 0.4% BSA, 2% B27, 20 ng/ml bFGF, 20 ng/ml EGF, and 5 μg/ml insulin). Mammospheres were passaged every 4 days. The numbers of secondary generation spheres (at least 50 μm in diameter) were counted using microscope (Nikon, Japan) fitted with graticule at 100× magnification. The percentage of mammosphere-forming efficiency was calculated, as previously described^21^.

### Lentivirus-mediated interference

The engineering cell lines with sh-lnc408, lnc408, sh-SP3, sh-CBY1, or CBY1 were established by lentivirus-mediated infection, according to previous instructions^21^. For short hairpin RNAs (shRNA)-mediated interference, shRNAs against lncRNA-408, SP3, or CBY1 were designed with the Clontech RNAi Target Sequence Selector (http://bioinfo.clontech.com/rnaidesigner/sirnaSequenceDesignInit.do). At least three shRNAs were designed for each gene, and the two most effective shRNAs were used for subsequent research. For overexpression, full length of lncRNA-408 and CBY1 cDNAs were acquired from Ensemble database (http://asia.ensembl.org/). All the shRNA oligonucleotides and synthetic cDNA inserts were cloned into the pGLVH1/GFP vector (GenePharma, CHN). Breast cancer cells were transfected as RNAi consortium (http://www.broadinstitute.org/rnai/trc) described, stable cell lines were established after being screened with puromycin. The core sequences of shRNA are listed in Supplementary Table 1.

### Patients and sample collection and immunohistochemistry (IHC)

Human breast tumor samples were recruited from the First Affiliated Hospital of Chongqing Medical University, according to the approval of the Ethics Committee of Chongqing Medical University. All the involved patients were consented to take part in the study and to publish the results. None of them has received any radiotherapy or chemotherapy before the operation.

The immunohistochemical protocols were performed, as done previously^22^. In brief, tumor tissues were fixed with 10% formalin. Paraffin-embedded specimens were sectioned at 4 μm thickness. Tissue sections were incubated with specific primary antibody. To eliminate nonspecific staining, normal IgG was used as a negative control. Image-Pro plus 6.0 software (Media Cybernetics, USA) was employed to quantify the IHC staining. Data are presented as mean optical density (MOD: IOD/area).

### Real-time PCR

Total RNA was extracted using the TRIzol reagent (TAKARA, Japan), then subjected to reverse transcription reactions by using Prime Script RT reagent Kit (Takara Bio., CHN). cDNA was used as a template for quantitative real-time PCR using the SYBR Premix Ex Taq II Kit (Takara Bio., CHN). Sequence-specific primers are listed in Supplementary Table 2.

### Western blotting

Cells were crushed in RIPA lysis buffer (Beyotime, CHN), then quantified with BCA protein assay kit (Beyotime, CHN). Western blotting assay was performed, as done previously^23^. Briefly, cell proteins were separated by 6–12% SDS–PAGE gel, then the proteins were transferred to PVDF membrane. After incubated with primary antibody and HRP-conjugated secondary antibody sequentially, the membrane was visualized by the enhanced chemiluminescence system (Bio-Rad, Hercules, USA).

### Immunoprecipitation western blotting (IP-WB)

IP-WB was performed, as described previously^24^. Briefly, BT549 or HEK293T cells were lysed with lysis buffer (Beyotime, CHN) containing Triton X-100. In parallel, specific antibody or IgG control (Abcam, UK) was incubated with Protein G Dynabeads (Invitrogen, USA). Then cell lysates were incubated with antibody-conjugated beads overnight, after being washed with lysis buffer for five times, the beads were boiled in SDS sample buffer and the released proteins were resolved by SDS–PAGE. The immunoprecipitates were detected by western blotting with specific primary antibody.

### Flow cytometry and cell sorting

Breast cancer cells were incubated with APC-conjugated anti-human CD44 (BD, USA) and PE-conjugated anti-human CD24 (BD, USA) antibodies, followed by detecting with CytoFLEX (Beckman Coulter, USA). For cell sorting, labeled cells were sorted with Influx cell sorter (BD, USA).

### Immunofluorescence staining (IF) and RNA fluorescence in situ hybridization (RNA-FISH)

For IF staining, cells were plated on preprepared coverslips in a 24-well plate and cultured for 6 h, then fixed with 4% paraformaldehyde. After cells were incubated with specific primary antibody against CD44 or SOX2, the Cy3-labeled secondary antibody was used, then the coverslips were sealed by glycerol. The normal rabbit or mouse IgG was used as a negative control in this experiment.

RNA-FISH assay was carried out according to manufacturer’s instruction. Fluorescence-conjugated lnc408 probes were synthesized from Ribobio (www.ribobio.com, CHN). Generally, cells were prepared as IF, as described above. The coverslips were fixed with 4% paraformaldehyde and then hybridized with lncRNA-408 probes for overnight. After being eluted by different concentrations (1–4×) of sodium citrate buffer, the cells were washed with PBS. Photographs were captured by confocal microscopy (Leica, GER). U6 served as a nucleus control and 18S served as a cytoplasm control, cell nuclei were counterstained with DAPI.

### RNA pull down and RNA immunoprecipitation (RIP)

RNA pull-down assay was performed with Pierce™ Magnetic RNA–Protein Pull-Down Kit (Thermo Fisher, USA), according to the manufacturer’s instructions. Biotin-labeled lncRNA-408, antisense lncRNA-408 and control RNA probes were synthetized by GenePharma (www.genepharma.com). Briefly, labeled RNA probes were bound to streptavidin magnetic beads, then incubated with protein lysate of CSCs. The RNA-binding protein complexes were eluted and separated with SDS–PAGE. The gel was subjected to silver staining, followed by collection of the interesting bands for mass spectrometry.

RIP assay was done using Millipore EZ-Magna RIP RNA-binding protein immunoprecipitation kit (Millipore, USA). Generally, CSCs were collected and dissolved with RNase-free RIPA lysate (Beyotime, CHN). The protein A/G beads were precleared and incubated with SP3 antibody. Then the protein A/G beads were incubated with supernatants overnight. Total RNA was extracted from the RIP products, and lncRNA-408 enrichment was detected by qRT-PCR.

### Tumor xenografts

All experimental procedures were approved by the animal care ethics committees at Chongqing Medical University. BT549/sh-lnc408 and its control mammosphere cells (1 × 10^3^–1 × 10^5^ cells per mice); BT549/non-CSCs (1 × 10^6^ cells per mice) transfected with lnc408, lnc408 combined with CBY1, or the control were subcutaneously injected into mammary fat pad of aged 4–6-week female nude mice (*n* = 10 per group). Tumor volume (*V* = length × width^2^ × 0.5) were measured every 4 days. The mice were raised under standard conditions as the institutional guidelines for animal care suggested. The mice were euthanized at the fifth week after xenografts for further research.

### Statistical analysis

Data were analyzed in SPSS standard version 19.0 or GraphPad Prism 7. Student’s *t* test was utilized for single comparisons between two groups, and ANOVA followed by the Student–Newman–Keuls multiple comparison test was conducted for a comparison between multiple groups. The data are presented as mean ± SD from at least three independent experiments. *P* < 0.05 was considered to indicate statistical significance.

## Results

### Lnc408 is highly expressed in BCSCs

**Fig. 1:**
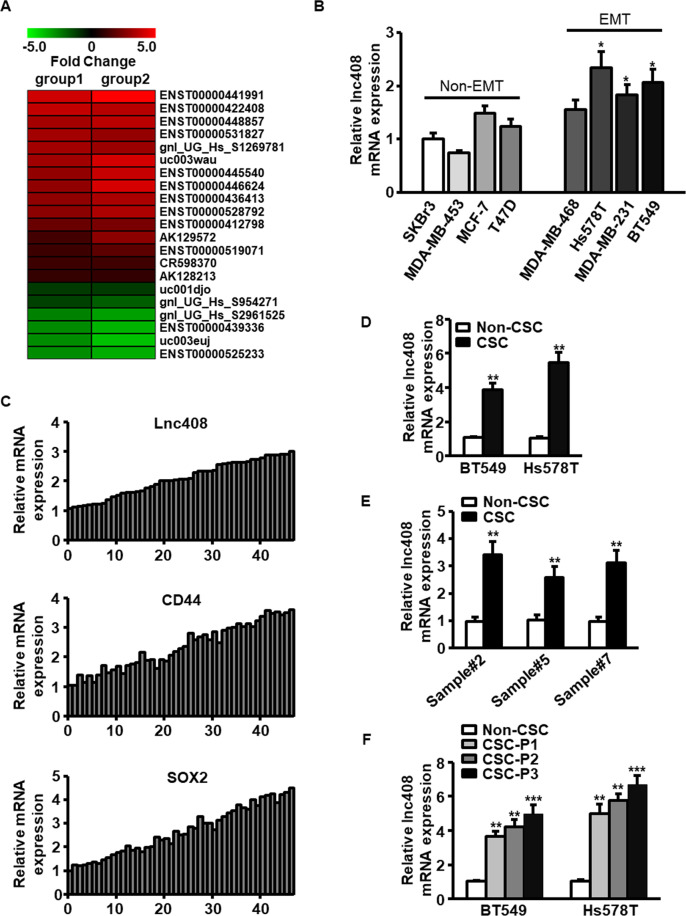
Lnc408 is upregulated in BCSCs. **A** The heat map showed the major dysregulated stemness-associated lncRNAs in BCSCs derived from epithelial or mesenchymal MCF-7 cells. The red or green dots indicated upregulated or downregulated lncRNAs, respectively. Data are shown as relative fold changes (>2.0) of MCF-7/Twist vs MCF-7/Vector. **B** Lnc408 expressions were proved by qRT-PCR analysis in non-EMT and EMT BC cells (SKBr3 as a control group, *P < 0.05). **C** qRT-PCR was used to assess the expression levels of lnc408, CD44, and SOX2 in BC tissues (*n* = 50). **D**, **E** qRT-PCR was conducted to determine the abundances of lnc408 in BCSC derived from BC cell lines and clinical BC samples (**P < 0.01). **F** Lnc408 expression was determined by qRT-PCR in different passage of BCSCs (P1 to P3; **P < 0.01, ***P < 0.001). For qRT-PCR assays, all data are shown as means ± SD.

Our previous study revealed that epithelial–mesenchymal transition (EMT) leads to enhanced stemness characteristic of breast cancer cells^21^. Using lncRNA array, we identified a series of dysregulated lncRNAs in BCSCs, 21 of which were stemness-associated lncRNAs (Fig. 1A), and some of these lncRNAs were further validated by qRT-PCR (Fig. S1A). Among these lncRNAs, ENST00000422408, termed lnc408 here after, was one of the most highly expressed lncRNAs in BCSCs. To confirm the relationship between lnc408 expression and BCSCs, lnc408 levels were evaluated in CSCs derived from various breast cancer cells, and we found that higher level of lnc408 was in EMT breast cancer cell lines (MDA-MB-468, Hs578T, MDA-MB-231, and BT549) than that in non-EMT cell lines (SKBr3, MDA-MB-453, MCF-7, and T47D). In addition, lnc408 expression was positively correlated with the protein level of stemness marker (e.g., CD44 and SOX2) in both breast cancer cells and clinical samples (Fig. S1B and Fig. 1C).

CD44^+^/CD24^−^ has been widely used as BCSC surface markers, thus we sorted CD44^+^/CD24^−^ subpopulation (termed as BCSCs) from various breast cancer cells and clinical tumor samples, and other remaining subpopulations (CD44^+^/CD24^+^, CD44^−^/CD24^+^, CD44^−^/CD24^−^) were termed as non-BCSCs. The higher level of lnc408 was further confirmed in these CD44^+^/CD24^−^ subpopulation compared with their non-BCSCs (Figs. 1D, E). Moreover, the gradually elevated lnc408 was observed in the BCSC spheres from first to third generation (Fig. 1F). Kaplan–Meier survival curves showed that patients with high expression of lnc408 had a shorter overall survival than those with low expression of lnc408 (Fig. S1C). These data demonstrate that lnc408 is highly expressed in BCSCs.

### Lnc408 is required for self-renewal maintenance of BCSCs

**Fig. 2:**
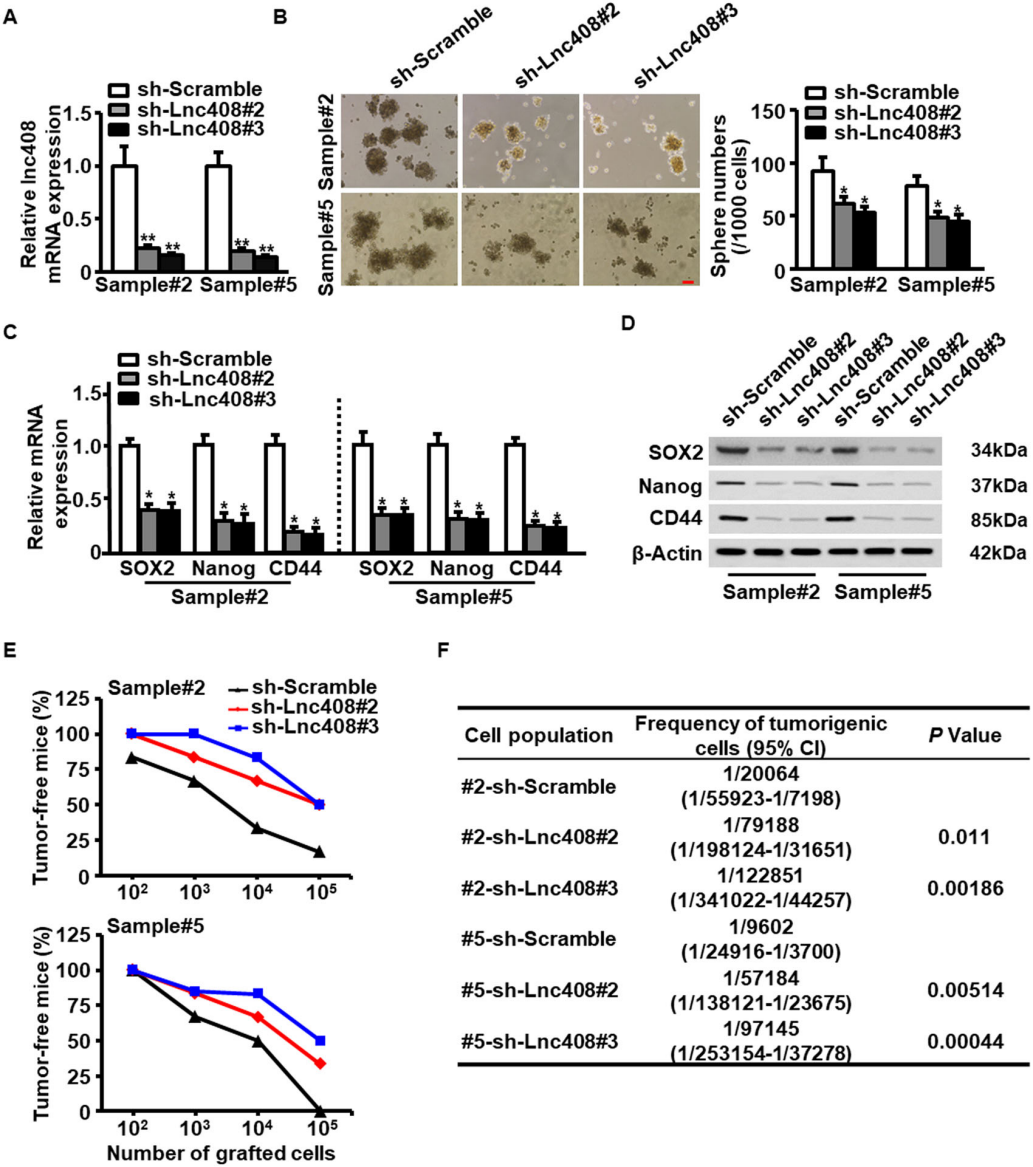
Lnc408 is required for the self-renewal maintenance of BCSCs. **A** Lnc408 was efficiently silenced by two-independent shRNAs (#2 and #3) in primary BC cells (**P < 0.01). **B** Mammosphere-forming capacities in primary BC cells with silenced lnc408 were evaluated by suspended culture, and representative images were shown. The right panel represents the statistical results of mammosphere numbers as means ± SD (*P < 0.05; scale bar, 100 μm). **C**, **D** BCSC stemness markers (SOX2, Nanog, and CD44) were assessed in lnc408-depleted cells by qRT-PCR (**C**) and western blotting (**D**) (*P < 0.05). **E** Lnc408-depleted or control primary BC cells were transplanted into mammary fat pads of nude mice in tenfold serial dilution manner. Tumor initiation and tumor sizes were monitored over 2 months (*N* = 6 for each group). **F** Frequency of tumorigenic cells in lnc408-depleted and scramble primary B cells was analyzed by extreme limiting dilution analysis (#2 clinical sample#2, #5 clinical sample#5, CI confidence interval).

To determine the role of lnc408 in BCSC self-renewal, we silenced lnc408 by separately transfecting two lentivirus-mediated shRNAs into breast cancer cells and primary breast cancer cells derived from clinical samples (Fig. 2A and Fig. S2A). Lnc408 knockdown dramatically reduced sphere formation of breast cancer cells and primary breast cancer cells (Fig. 2B and Fig. S2B). Moreover, the mRNA (Fig. 2C and Fig. S2C) and protein (Fig. 2D and Fig. S2D) levels of representative breast cancer stemness markers, including SOX2, Nanog, and CD44 were notably decreased in the lnc408-knocked down cells compared to their scrambled control cells (sh Ctrl). In line with this, lnc408 knockdown attenuated CD44 and c-Myc expressions in the BCSC spheres checked by IF staining (Fig. S2E). Subsequently, we explored the effects of lnc408 on tumor-initiating capacity using lnc408 wild-type and knocked down primary breast cancer cells isolated from breast cancer tumors, which were subcutaneously injected into mammary fat pad of nude mice. Lnc408 knockdown led to the significant reduce of tumor burden compared with the control primary cancer cells, as detected by a limiting dilution xenograft analysis (Fig. 2E). Furthermore, lnc408 knockdown apparently decreased tumorigenic cell frequency (Fig. 2F). Taken together, these data indicate that lnc408 is required for the maintenance of self-renewal capability of BCSCs.

### Lnc408 endows non-BCSCs to acquire stemness property

**Fig. 3:**
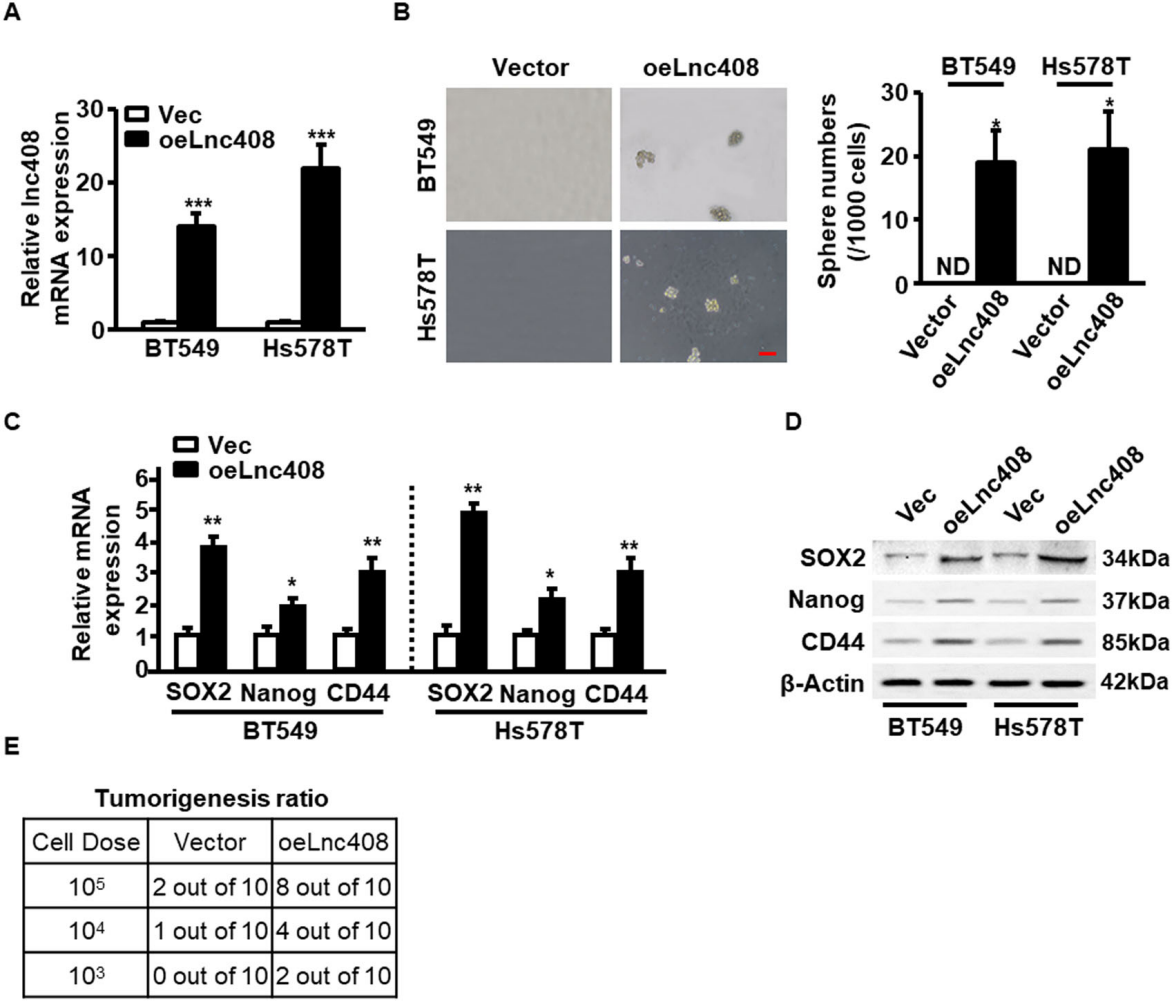
Lnc408 endow non-CSCs to acquire stemness characteristics in breast cancer. **A** The expression of ectopic lnc408 was confirmed in non-stemness characteristic breast cancer cells (BT549 and Hs578T; ***P < 0.001; Vec control vector, oeLnc408 overexpress of ectopic lnc408). **B** Lnc408 overexpression endowed non-CSC breast cancer cells with CSC characteristics, checked by mammosphere formation capacity. The right panel represents the statistical results of mammosphere numbers as means ± SD (*P < 0.05; ND none detected; scale bar, 100 μm). **C**, **D** Breast cancer stemness markers (SOX2, Nanog, and CD44) were detected in lnc408-overexpressed cells by qRT-PCR (**C**) and western blotting (**D**) (*P < 0.05, **P < 0.01). **E** Non-CSCs transfected with ectopic Lnc408 or control vector were transplanted into mammary fat pads of nude mice in different cell dose (1 × 10^3^–1 × 10^5^ cells/each mouse), tumorigenicity of nude mice was measured during the experimental period (over 3 months; *N* = 10 for each group).

To further clarify the relationship between lnc408 and BCSCs stemness, ectopic lnc408 was stably transfected into non-CSC subpopulation derived from breast cancer cell lines and primary cancer cells (Fig. 3A and Fig. S3A). Compared with non-BCSCs, lnc408 overexpression can restore the non-BCSCs with the sphere formation abilities (Fig. 3B and Fig. S3B). Correspondingly, ectopic lnc408 obviously augmented both mRNA (Fig. 3C and Fig. S3C) and protein (Fig. 3D and Fig. S3D) expressions of breast cancer stemness markers (e.g., SOX2, Nanog, and CD44). Moreover, the effect of ectopic lnc408 on tumorigenesis of non-BCSC population was assessed using limiting dilution experiments in vivo. As few as 1 × 10^3^ of lnc408-overexpressing BT549 cells was sufficient for tumor initiation (2/10 vs 0/10), 1 × 10^4^ of lnc408-overexpressing BT549 cells led to more tumor initiation (4/10 vs 1/10), and injection of 1 × 10^5^ of lnc408-overexpressing BT549 cells resulted in tumorigenesis in most of mice in comparison of non-BCSC cells (8/10 vs 2/10; Fig. 3E), suggesting that lnc408 can enable non-CSCs to acquire stemness characteristic in breast cancer.

### Lnc408 suppresses the transcription of CBY1 to maintain BCSCs self-renewal

**Fig. 4:**
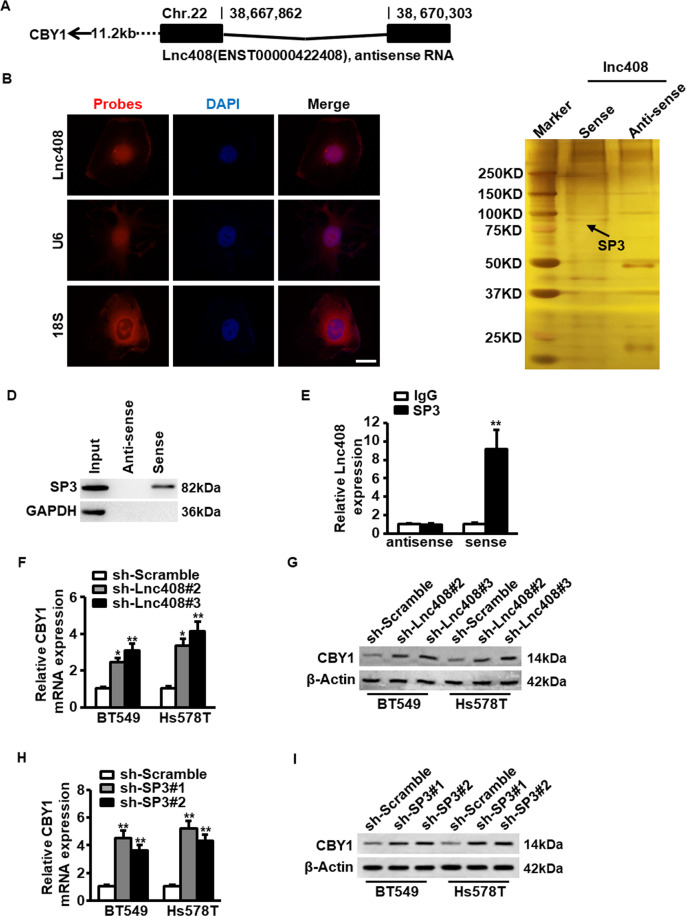
Lnc408 regulates the expression of target gene CBY1. **A** Schematic annotation of lnc408 genomic locus on chromosome 22. Black rectangles represent exons. **B** Intracellular localization of lnc408 was visualized in BC cells by RNA-FISH assays. Representative images of lnc408 in BT549 cells are shown. U6 served as a nucleus control and 18S as a cytoplasm control; cell nuclei were counterstained with DAPI (scale bar, 10 μm). **C** Full length of lnc408 transcripts (sense), antisense transcripts and control probe were labeled with biotin, then RNA-pull down was performed using nuclear extracts of mammospheres derived from BT549 cells. The interesting bands were followed by mass spectrometry. The arrow indicates the target of SP3. **D** The lnc408-binding protein SP3 in RNA-pull down precipitates (as carried out as in **C**, GAPDH as a negative control) was further confirmed by western blotting. **E** The interaction between lnc408 and SP3 was verified by an RNA immunoprecipitation (RIP) assay (**P < 0.01). Data are shown as means ± SD. **F**, **G** The mRNA or protein expression of CBY1 was determined by qRT-PCR (**F**) and western blotting (**G**) in lnc408-silenced BCSCs, respectively (*P < 0.05, **P < 0.01). **H**, **I** The mRNA and protein expressions of CBY1 were determined by qRT-PCR (**H**) and western blotting (**I**) in SP3-knocked down BCSCs, respectively (**P < 0.01).

Next, we asked how lnc408 involves in stemness maintenance of BCSC. It has been reported that lncRNAs can act in cis-regulatory effects to regulate neighboring gene expression^25^. We found that lnc408 was an antisense lncRNA composed of two exons and spanning 1.08 kilobases (kb), and locating on chromosome 22 near to chibby 1 (CBY1) gene (Fig. 4A). Furthermore, lnc408 was confirmed to be a lncRNA with no coding potentiality (Fig. S4A and Fig. S4B), and mainly distributed in nuclei of breast cancer cells checked by RNA-FISH assay (Fig. 4B).

Since lncRNAs could exert their functions through interacting with proteins to regulate gene expression in the nuclei^26^, an RNA pull-down assay with biotin-labeled lnc408 was performed to seek the potential lnc408-associated proteins. SP3 was identified to bind with lnc408 in BCSCs (Fig. 4C, D), and the interaction of lnc408 with SP3 was further validated by immunoprecipitation (RIP; Fig. 4E). In addition, we confirmed that lnc408 knockdown promoted CBY1 mRNA (Fig. 4F) and protein (Fig. 4G) expression in BCSCs. Similarly, efficient silence of SP3 (Fig. S4C, D) led to the enhanced mRNA (Fig. 4H) and protein (Fig. 4I) levels of CBY1, which is consistent with the finding that SP3 could serve inhibitory transcription function^27,28^. These data suggest that lnc408 recruits SP3 to suppress the transcription of CBY1.

**Fig. 5:**
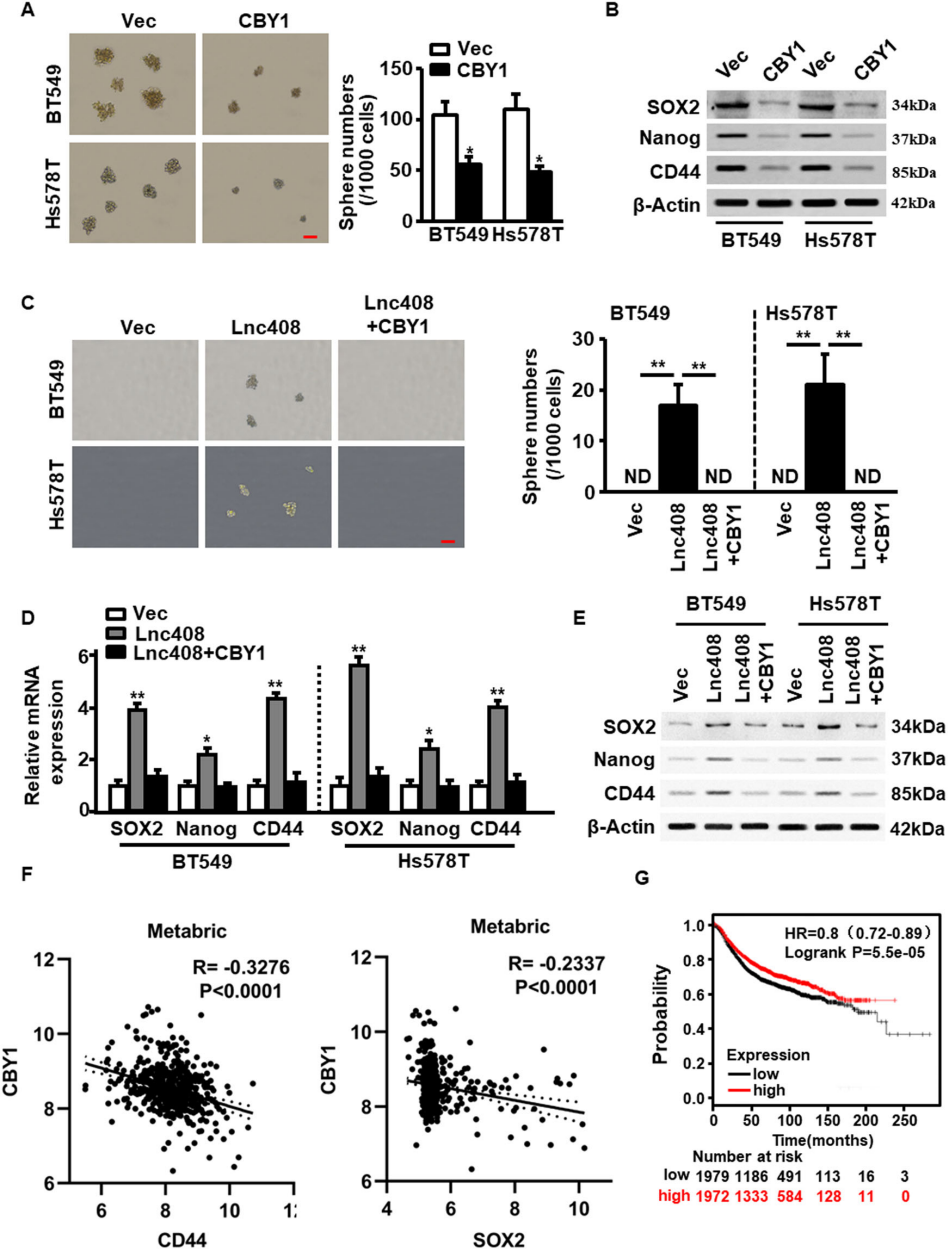
Lnc408 suppresses the expression of CBY1 to regulate BCSC self-renewal and stemness maintenance. **A** Ectopic CBY1 was transfected into BC cells to check the mammosphere-forming capacity of CSCs, and representative pictures of mammospheres are shown. The right panel represents the statistical results of mammosphere numbers as means ± SD (*P < 0.05; scale bar, 100 μm). **B** Breast cancer stemness markers (SOX2, Nanog, and CD44 were assessed in CBY1-overexpressed BCSCs by western blotting. **C** The non-CSC-like BC cells were transfected with control vector, lnc408 or lnc408 combined with CBY1, the mammosphere formation capacities were assessed in serum-free suspended culture. The right panel represents the statistical results of mammosphere numbers as means ± SD (**P < 0.01; ND none detected; scale bar, 100 μm). **D**, **E** The mRNA and protein levels of CSC markers (SOX2, Nanog, and CD44) were determined by qRT-PCR (**D**) and western blotting (**E**), respectively (*P < 0.05, **P < 0.01). **F** Pearson correlation analysis of CBY1 and CD44, CBY1 and SOX2 in 474 breast cancer tissues based on Metabric database. **G** Kaplan–Meier survival analysis to show the BC patients with low CBY1 expression (*N* = 3851) had poor prognosis, using TCGA database.

To investigate the role of CBY1 in the self-renewal of BCSC, ectopic CBY1 was efficiently transfected into BCSCs (Fig. S5A, B). As shown in Fig. 5A, CBY1 overexpression markedly reduced sphere number and their average diameter. Correspondingly, ectopic CBY1 significantly decreased SOX2, Nanog, and CD44, the representative breast cancer stemness marker expressions both in mRNA (Fig. S5C) and protein (Fig. 5B) levels, implying that CBY1 has the ability to inhibit the stemness of BCSCs. To further verify the function of CBY1 in stemness maintenance of BCSCs, CBY1 was transfected into non-BCSC with ectopic lnc408, and mammosphere formation and CSC-associated markers were assessed. As shown, the ectopic lnc408-induced mammosphere formation potentials (Fig. 5C) and stemness-related protein expressions (Fig. 5D, E) were notably decreased by CBY1. Besides, the expression of CBY1 was negatively related with the levels of CD44 and SOX2, two of known stemness biomarkers, in our cohort of clinical samples (Fig. S5D), these findings were further confirmed in large samples downloaded from Metabric database (Fig. 5F). Indeed, the breast cancer patients with high level of CBY1 had a higher overall survival in comparison with these with low level of CBY1 (Fig. 5G). Overall, lnc408 may involve in maintenance of BCSCs by inhibiting CBY1.

### Lnc408-mediated decrease of CBY1 activates Wnt/β-catenin signaling

**Fig. 6:**
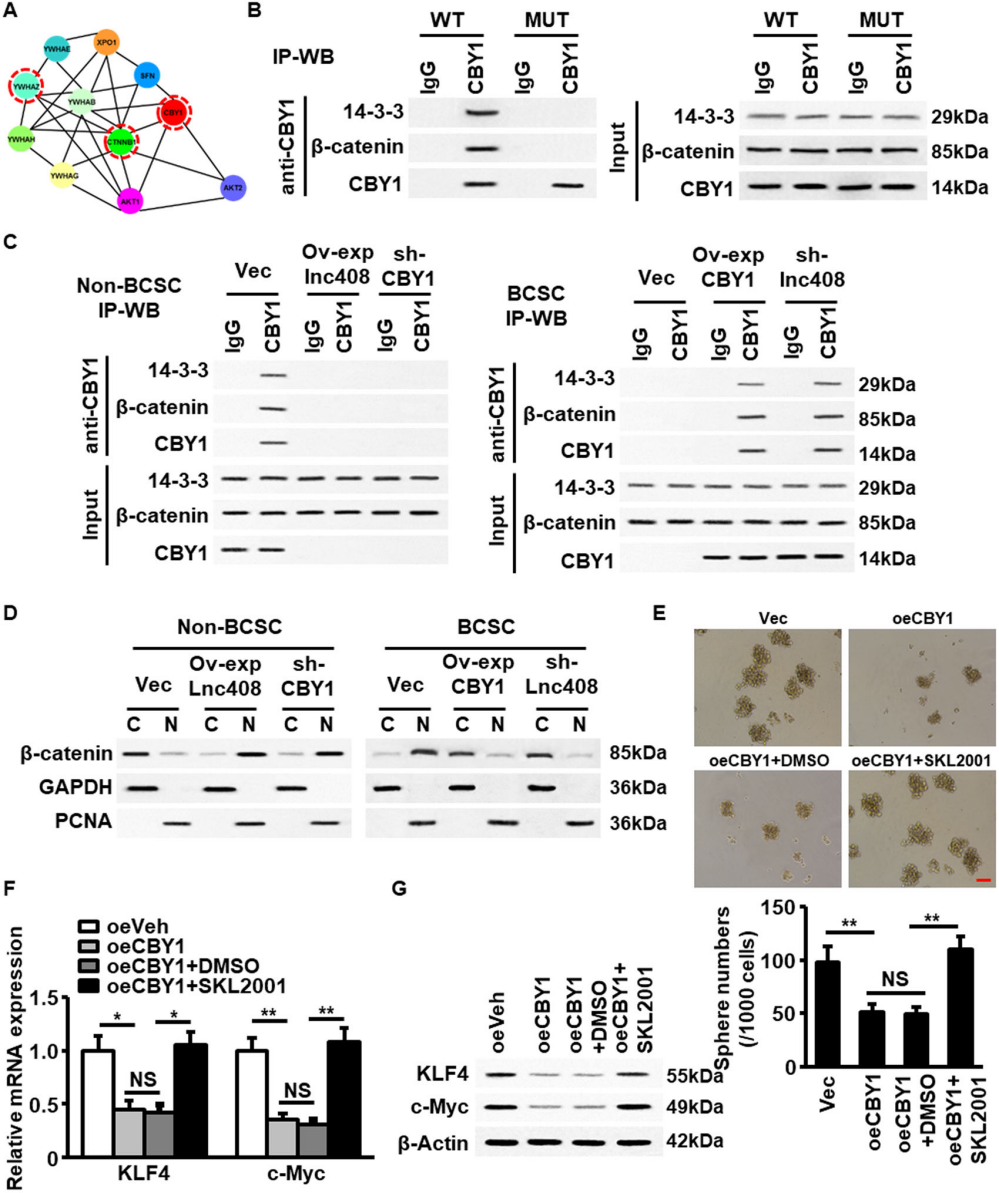
Lnc408-mediated CBY1 decrease triggers WNT signaling and promotes self-renewal and stemness maintenance of BCSCs. **A** A protein networks showing the potential interaction among 14-3-3 (YWHAZ), β-catenin (CTNNB1), and CBY1. **B** HEK293T cells were transfected with an expression vector encoding wild-type CBY1 (WT) or mutant CBY1 (S20A). Immunoprecipitation and western blotting were conducted with antibodies against 14-3-3, β-catenin, and CBY1 to detect the interaction of CBY1, 14-3-3, and β-catenin. **C** IP-WB was used to detect the ternary complex of CBY1, 14-3-3, and β-catenin in non-CSC derived from of BT549, non-CSC transfected with ectopic lnc408 (oelnc408), and non-CSC with silenced CBY1 (sh-CBY1); or in CSC derived from BT549, CSC transfected with ectopic CBY1 (oeCBY1), and CSC with silenced lnc408 (sh-lnc408). **D** Western blotting to check the cellular distribution of β-catenin as in **C** (C cytoplasm, N nucleus). GAPDH served as a cytoplasm control, and PCNA as a nucleus control. **E** The mammosphere-forming abilities of BT549, BT549 transfected with CBY1 (oeCBY1), or BT549/ocCBY1 cells treated with or without SLK2001 (WNT/β-catenin agonist, 10 μM) were tested under suspended culture. The down panel shows the statistical results of mammosphere numbers as means ± SD (**P < 0.01; NS none significance; scale bar, 100 μm). **F**, **G** The mRNA and protein expressions of breast cancer pluripotent factors (KLF4, c-Myc) were determined by qRT-PCR (**F**) and western blotting (**G**), respectively (*P < 0.05; **P < 0.01; NS none significance).

Subsequently, we wondered how lnc408-mediated CBY1 involves in regulating the stemness of BCSCs. By using bioinformatics analysis, we found CBY1 could interact with 14-3-3, AKT, β-catenin, and other proteins (Fig. 6A). It has been reported that the phosphorylation of CBY1 by ATK lead to an activated CBY1, which binds with 14-3-3 and β-catenin to form a ternary complex, and causing β-catenin to be pumped out of nuclei^29^. Agreeing with the previous findings, we confirmed that CBY1 could interact with 14-3-3 and β-catenin to form a ternary complex, mutation of CBY1 at 20 sites from serine into alanine (mutant CBY1 S20A) caused the disintegration of the complex (Fig. 6B) in 293T cells. Indeed, the trimer of CBY1/14-3-3/β-catenin was mainly existed in non-BCSCs rather than in BCSCs, which could be abrogated when either ectopic lnc408 was transfected into non-BCSC or CBY1 was silenced in non-BCSC (Fig. 6C, left panel). However, restoration of CBY1 expression or knockdown of lnc408 in BCSC facilitated the ternary complex formation (Fig. 6C, right panel). Consistently with the ternary complex formation in non-BCSCs or disintegration in BCSCs, higher level of cytoplasmic β-catenin than nuclear β-catenin was detected in non-BCSCs, the distribution of cytoplasmic or nuclear β-catenin in non-BCSC could be reversed by overexpression of lnc408 or knockdown of CBY1 (Fig. 6D, left panel); however, the lower level of cytoplasmic β-catenin and higher level of nuclear β-catenin in BCSCs could also be reversed by ectopic CBY1 or silence of lnc408 (Fig. 6D, right panel). Correspondingly, CBY1-mediated the ternary complex formation and nuclear β-catenin accumulation were closed with the change of mammosphere formation capability in BCSC derived from BT549 cells (Fig. 6E), in which ectopic CBY1 induced decrease of nuclear β-catenin blunted mammosphere formation, SKL2001 (an agonist of Wnt signaling) treatment rescued the spheres formation ability (Fig. 6E). In addition, previous study has found that stemness-related gene c-Myc and Klf4 were at downstream of Wnt/β-catenin signaling in bovine embryonic stem cells (ESCs)^30^. Similarly, the same trend of stemess-related gene expression, including c-Myc and Klf4 was observed in these BCSCs (Fig. 6F, G). These data demonstrate that lnc408-mediated decrease of CBY1 stimulates Wnt/β-catenin signaling and acts a pivotal role in stemness maintenance of BCSCs.

### Lnc408/CBY1 axis facilitates BCSC tumorigenesis in vivo

**Fig. 7:**
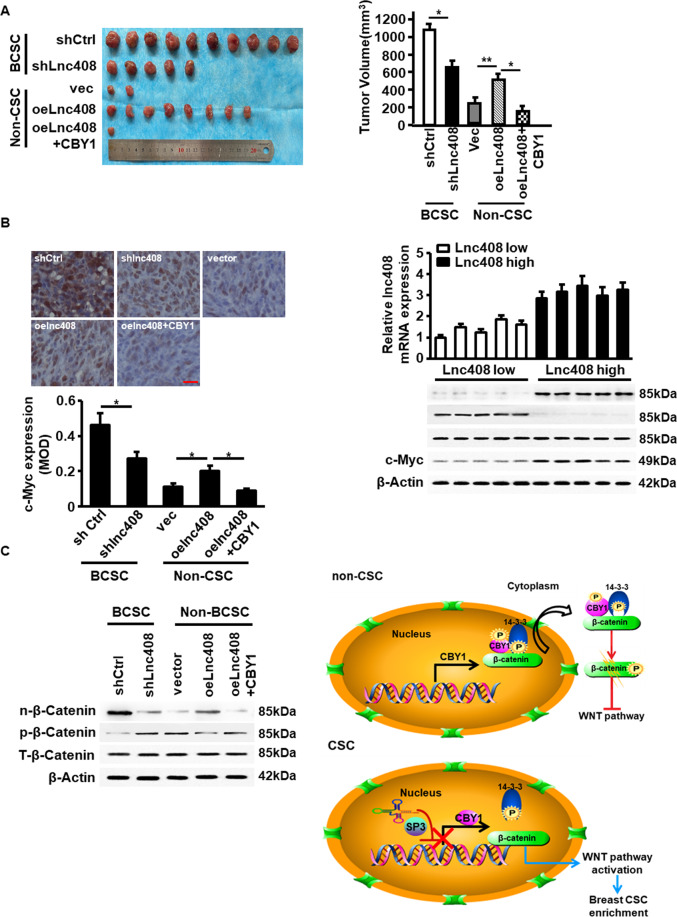
Lnc408/CBY1 axis promotes tumorigenesis of BCSCs in vivo. **A** The tumor initiation and tumor sizes of each group (*P < 0.05, **P < 0.01). **B** Representative pictures of IHC staining of c-Myc proteins (upper panel) and c-Myc mRNA levels (down panel) in each group (*P < 0.05; scale bar, 50 μm). **C** Protein levels of nuclear, phosphorylated, and total β-catenin in tumors were determined by western blotting. **D** Clinical samples were divided into lnc408 high and low expression groups according to lnc408 expression levels, and subjected to western blotting to assess nuclear β-catenin, total β-catenin, and c-Myc expressions. **E** A schematic model to illustrate lnc408 molecular functions in BCSCs. Lnc408 recruits SP3 to impair CBY1 transcription, the reduced CBY1 lose or significantly incapacitated its ability to form complex with 14-3-3 and β-catenin in CSCs, which notably mitigate the transportation of β-catenin into cytoplasm, to lead an accumulation of nucleus β-catenin, thus triggers the WNT/β-catenin signaling to maintain BCSCs stemness.

Lastly, the effect of lnc408/CBY1 axis on tumorigenesis and tumor growth was assessed in vivo by injection of lnc408-knocked down BCSCs and lnc408-overexpressing non-BCSCs into female nude mice. As expectedly, in comparison with BCSC with high level of endogenous lnc408, knockdown of lnc408 notably decreased tumorigenesis and tumor growth; while ectopic lnc408 in the non-BCSCs significantly promoted tumorigenesis and increased tumor growth, and the lnc408-droved tumor initiation was severely blunted by reexpression of CBY1 in non-BCSC (Fig. 7A). The changes of c-MYC protein, another known stemness biomarker, were detected in the corresponding tumor tissues (Fig. 7B). Meanwhile, nuclear β-catenin protein, the downstream target of lnc408/CBY1 axis, was significantly increased in the tumor derived from injected BCSC (BCSC/shCtrl) compared to the tumor from injected non-BCSCs (non-BCSC/Vec), and it was reduced in tumor derived from lnc408-silenced BCSCs (Fig. 7C). The mice injected with lnc408-overexpressing non-BCSC had high rate of tumor initiation (Fig. 7A), which was compatible with the increased expression of nuclear β-catenin in the tumor (Fig. 7C); the mice injected with non-BCSC transfected with both ectopic lnc408 and CBY1 (non-BCSC/lnc408/CBY1) just had few tumor initiation (Fig. 7A), due to lowest nuclear β-catenin, which accompanied with high level of phosphorylated cytoplasm β-catenin, in the tumor (Fig. 7C), suggesting an essential role of lnc408/CBY1 axis in promoting tumor initiation of BCSCs via regulation of β-catenin signaling. To expand this finding, we assessed the lnc408 expression and the corresponding nuclear β-catenin and c-Myc protein levels in clinical tumor tissues. As shown in Fig. 7D, the enhanced nuclear β-catenin (n-β-catenin) and c-MYC (the representative stemness-related marker protein) accompanied with decreased phosphorylated cytoplasm β-catenin were detected in the randomly selected lnc408 high expressed tumors in comparison with the lnc408 low expressed tumors (Fig. 7D). In conclusion, in non-BCSCs, CBY1 forms ternary complex with 14-3-3 and β-catenin to result in the complex being bumped out of nucleus and the degradation of β-catenin; in BCSCs, lnc408 recruits SP3 to cause the transcription depression of CBY1, which prevents the formation of CBY1/14-3-3/β-catenin complex, and β-catenin is accumulated in the nucleus to promote the self-renewal and stemness of BCSCs (Fig. 7E).

## Discussion

CSCs, also termed as tumor-initiating cells, represent a minor subpopulation of cancer cells with intrinsic self-renewal and heterogeneous differentiation in most solid tumors. CSCs in breast cancer (BCSCs) are considered to be the leading cause in the relapse and metastasis of cancer. The treatment strategy targeting CSCs may be more effective. In recent years, CD44^+^/CD24^−^ subpopulations have been identified as BCSCs. However, little is known about the BCSCs biology details, especially in its self-renewal and stemness maintenance. In this study, we confirmed a novel lncRNA termed lnc408 plays a critical role in the self-renewal and maintenance of BCSCs.

Accumulating evidence shows that lncRNAs aberrantly expressed in various human diseases, especially in malignancies, such as breast cancer, lung cancer, prostate cancer, and bladder cancer^20,31,32^. Nonetheless, there are still many novel lncRNAs need to be discovered and their biological function to be unraveled. As an oncogene or tumor suppressor, lncRNAs perform important functions in breast cancer through regulating gene expression^16,33^. However, most of studies mainly focused on tumor proliferation, migration, and apoptosis^34,35^. The functions of lncRNA on CSC’s self-renewal remain largely unknown. In our study, we identified a novel lncRNA, lnc408, which is highly expressed in BCSCs and maintains stemness of BCSCs.

The way of lncRNA exerts the function mainly depends on its subcellular localization. Cytoplasmic lncRNA, could pare with other RNAs to act as a ceRNA; it could also interact with proteins to regulate the stability of mRNA or prevent proteins from degradation. Whereas nuclear lncRNA usually combines with chromatin-remodeling complexes to regulate its target gene expression at the transcriptional level, serves as epigenetic modifier^36^. In this study, we confirmed that lnc408 mainly distributed in the nucleus of BCSCs and suppressed CBY1 expression in cis-regulatory pattern through recruitment of SP3 transcription factor. This is consistent with previous findings that SP3 was a gene repressor specific protein in cocaine-induced transcriptional regulation^27,28,37^. Moreover, it has been reported that SP3 promoted lncRNA TINCR transcription to induce apoptosis and autophagy in cutaneous squamous cell carcinoma^38^. Here, we found that interaction between lnc408 and SP3 serves a new function in self-renewal of BCSCs, but the underlying mechanism needs to be further clarified.

CBY1 is called chibby 1, previous studies have demonstrated that CBY1 is downregulated in HCC, chronic myeloid leukemia, and colon carcinoma^39–41^. In line with this, our data show that CBY1 is a tumor suppressor associated with good prognosis in breast cancer. In addition, it has been reported that CBY1 facilitates cardiomyocyte differentiation of murine ESCs or suppresses aerobic glycolysis and proliferation of nasopharyngeal carcinoma^42,43^, both of them are based on the functional role of CBY1 as β-catenin antagonist, which means that CBY1 acts in concert with 14-3-3 proteins to facilitate export of nuclear β-catenin into cytoplasm^29^. Fortunately, our work revealed that lnc408 mediated a transcriptional suppression of CBY1, which impaired the ternary complex formation of 14-3-3/β-catenin/CBY1 to maintain a high level of β-catenin in the nucleus of BCSCs and played an essential role in the stemness maintenance of BCSCs through WNT/β-catenin signal pathway.

In conclusion, our study highlights a novel lnc408, which could recruit SP3 to suppress CBY1 transcription in the BCSCs. The loss of CBY1 prevents it from being an antagonist of β-catenin, and results in the self-renewal and stemness maintenance of BCSCs by activating WNT/β-catenin signal pathway. Our findings provide new insights into the treatment and prognosis of breast cancer.

## Supplementary information

Supplementary Figure 1

Supplementary Figure 2

Supplementary Figure 3

Supplementary Figure 4

Supplementary Figure 5

Supplementary Figure Legends

Supplementary Table S1

Supplementary Table S2
