# Supplementary figures and images for: A novel Lnc408 maintains breast cancer stem cell stemness by recruiting SP3 to suppress CBY1 transcription and increasing nuclear β-catenin levels
Source: Cell Death Dis. 2021 May 1;12(5):437. doi: 10.1038/s41419-021-03708-6 (PMC8088435; doi:10.1038/s41419-021-03708-6)

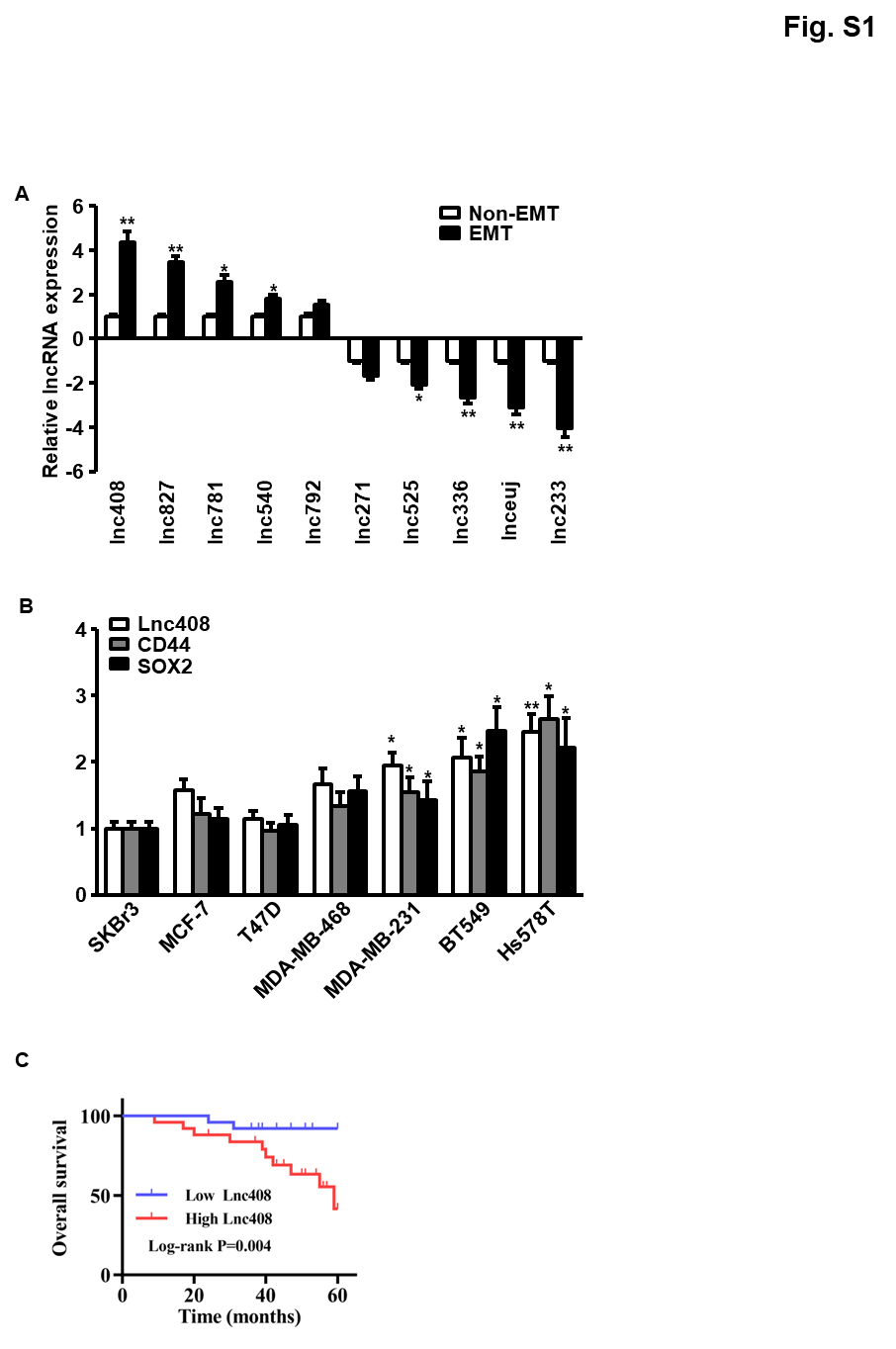

Supplement: Supplementary file 1 — Supplementary Figure 1 [file 41419_2021_3708_MOESM1_ESM.tif]

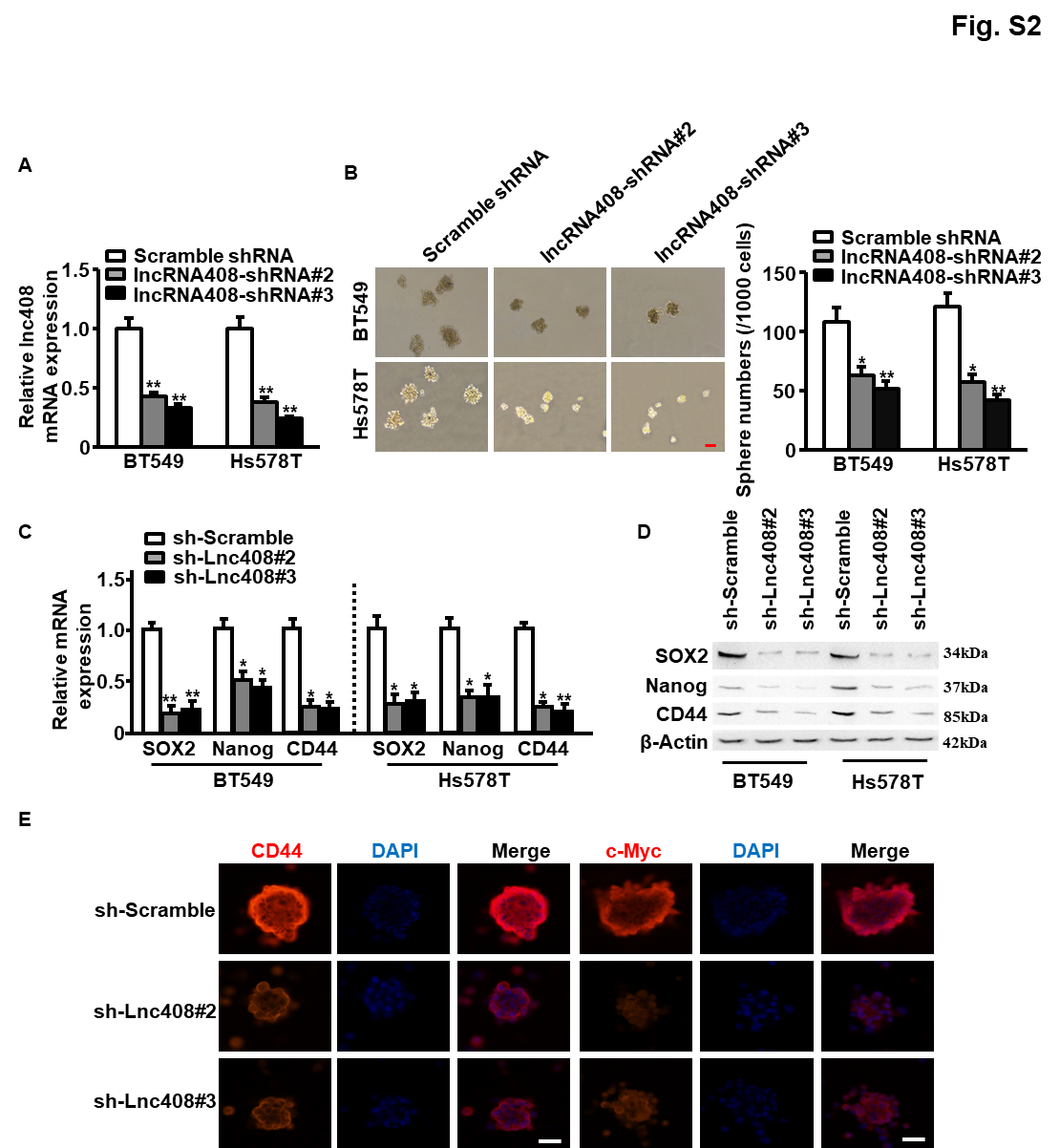

Supplement: Supplementary file 2 — Supplementary Figure 2 [file 41419_2021_3708_MOESM2_ESM.tif]

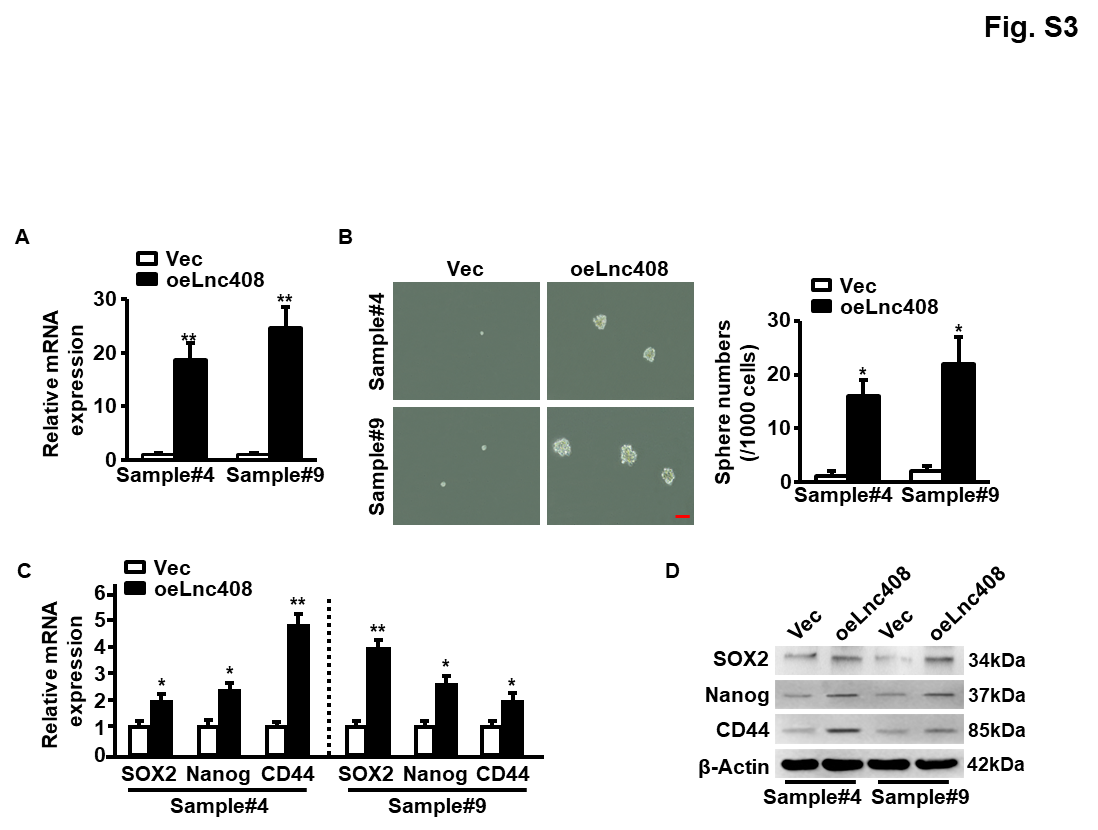

Supplement: Supplementary file 3 — Supplementary Figure 3 [file 41419_2021_3708_MOESM3_ESM.tif]

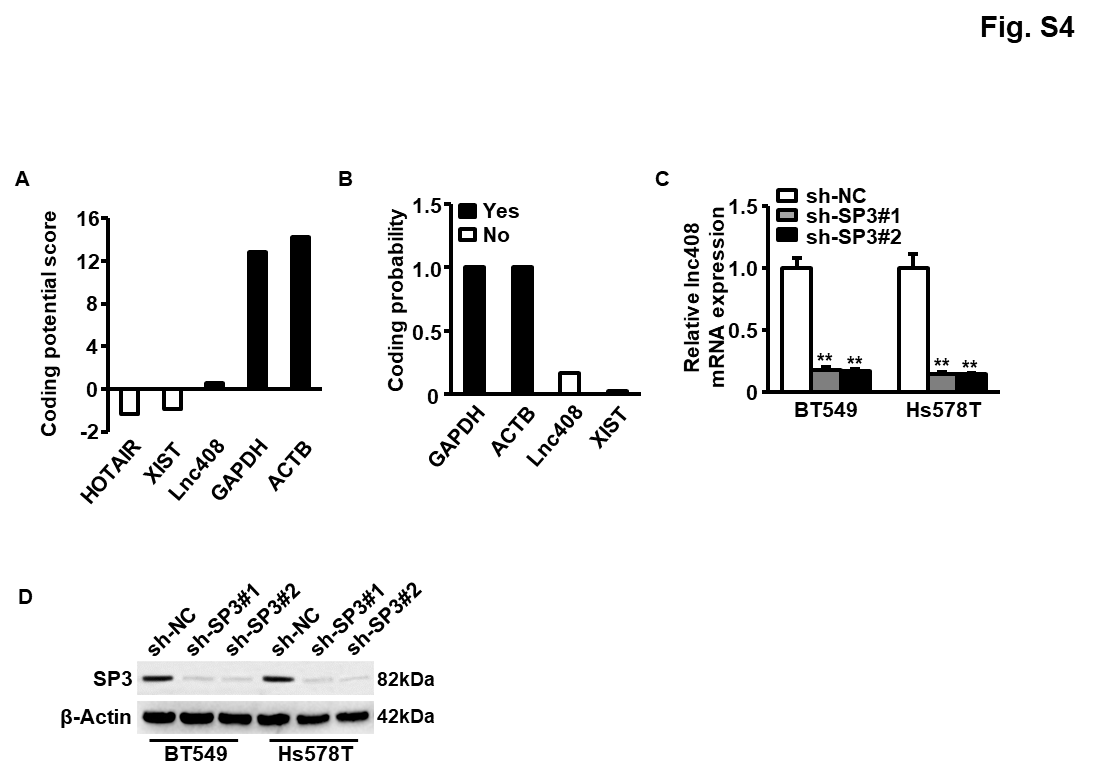

Supplement: Supplementary file 4 — Supplementary Figure 4 [file 41419_2021_3708_MOESM4_ESM.tif]

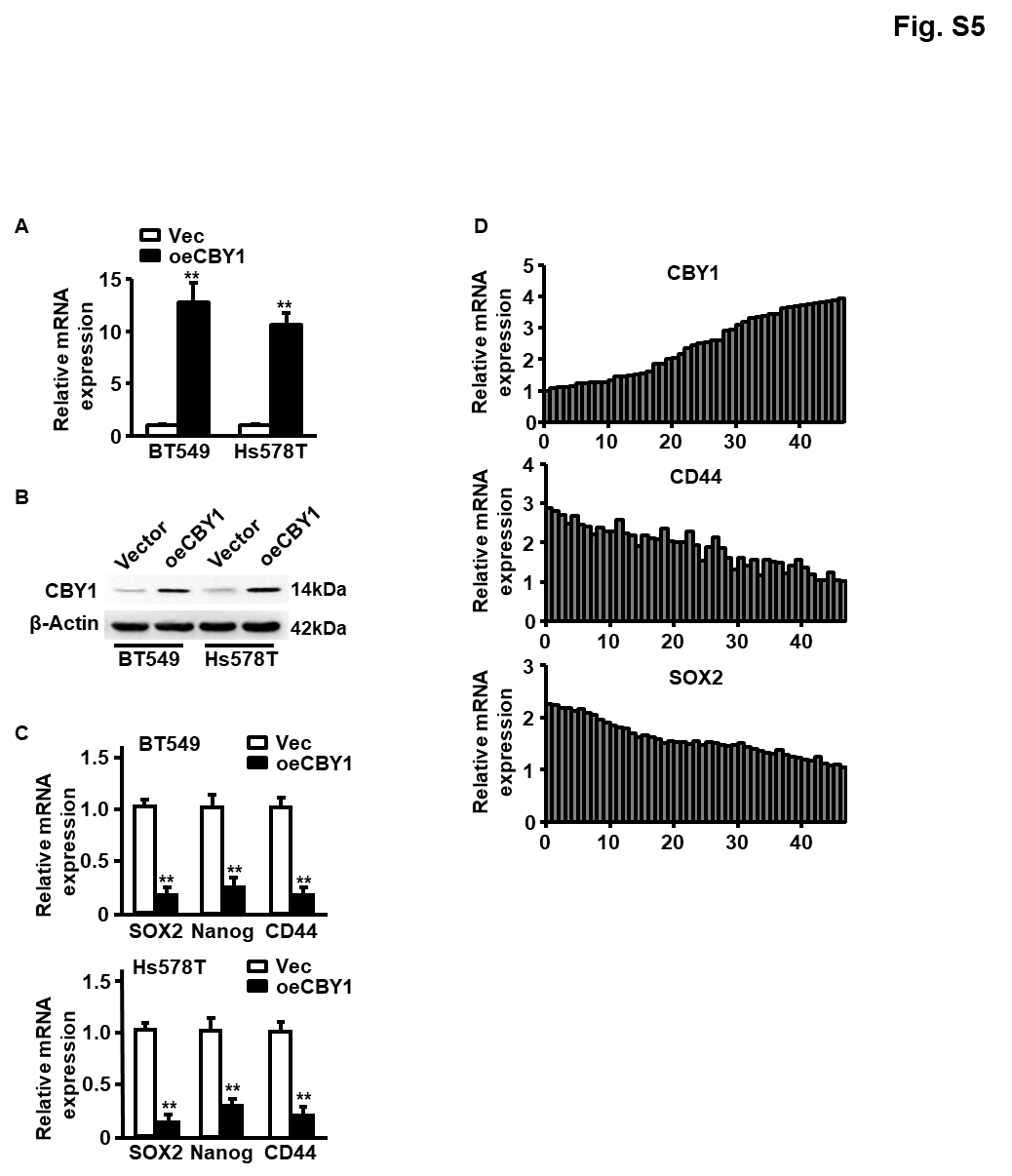

Supplement: Supplementary file 5 — Supplementary Figure 5 [file 41419_2021_3708_MOESM5_ESM.tif]
