# Supplementary material for: A novel Lnc408 maintains breast cancer stem cell stemness by recruiting SP3 to suppress CBY1 transcription and increasing nuclear β-catenin levels
Source: Cell Death Dis. 2021 May 1;12(5):437. doi: 10.1038/s41419-021-03708-6 (PMC8088435; doi:10.1038/s41419-021-03708-6)
Supplement: Supplementary file 6 — Supplementary Figure Legends [file 41419_2021_3708_MOESM6_ESM.docx]

**Supplementary Figure Legends**

**Figure S1 (related to Figure 1). Lnc408 is upregulated in BCSCs.**

(A) The levels of dysregulated lncRNAs were verified by qRT-PCR analysis in MCF-7/Vector and MCF-7/Twist CSCs (**P*<0.05, ***P*<0.01). (B) qRT-PCR was used to assess the levels of lnc408, CD44 and SOX2 in BCSC derived from different breast cancer cells(SKBr3 as a control group,**P*<0.05, ***P*<0.01).

**Figure S2 (related to Figure 2). Lnc408 is required for the self-renewal maintenance of BCSCs.**

(A) Lnc408 was silenced by two independent shRNAs (#2 and #3) targeting different exons in BT549 and Hs578T cells (***P*<0.01). (B) Lnc408 depletion resulted in an attenuated sphere-forming capacity in BC cells. The right panel shows the statistical results of mammosphere numbers as means ± SD (**P*<0.05, ***P*<0.01; Scale bar, 100 μm). (C-E) BCSC markers (CD44, SOX2 and Nanog,c-Myc) were measured in lnc408-depleted cells by qRT-PCR (C), western blotting (D) and immunoﬂuorescent staining (E) (**P*<0.05, ***P*<0.01). Cell nuclei were counterstained with DAPI (scale bar, 50 μm).

**Figure S3 (related to Figure 3). Lnc408 endows non-CSCs with stemness characteristics.**

(A) Ectopic lnc408 was transfected into in non-CSCs (CD44^-^/CD24^+/high^) isolated from primary tumor cells (clinical sample#4 and #9) (***P*<0.01; Vec, vector; oeLnc408, overexpress of lnc408). (B) Lnc408 overexpression endowed non-CSC derived from primary BC cells with mammosphere formation capacity. The right panel shows the statistical results of mammosphere numbers as means ± SD (**P*<0.05; ND, none detected; Scale bar, 100μm). (C, D) Ectopic lnc408 was transfected into non-CSCs isolated from primary tumor cells, and qRT-PCR (C) and western blotting (D) were used to evaluate the expressions of BCSC markers (SOX2, Nanog and CD44) (**P*<0.05, ***P*<0.01).

**Figure S4 (related to Figure 4). Lnc408 regulates the expression of target gene CBY1.**

(A, B) Coding potency of lncRNA408 sequence was analyzed using Coding Potential Calculator (A) or Coding Potential Assessment Tool (B). HOTAIR and XIST served as non-coding control, GAPDH and β-actin (ACTB) served as coding control. (C, D) SP3 mRNA (C) and protein expressions (D) were detected by qRT-PCR or western blotting in BT549 and Hs578T cells transfected with sh-SP3 or control shRNA, respectively. Data are given as mean ± SD (***P*<0.01).

**Figure S5 (related to Figure 5). Lnc408 suppresses the expression of CBY1 to regulate BCSCs self-renewal and stemness maintenance.**

(A, B) CBY1 mRNA (A) and protein levels (B) were determined by qRT-PCR or western blotting in BT549 and Hs578T cells transfected with CBY1 or control vector, respectively. Data are given as mean ± SD (***P*<0.01). (C) BCSC markers (SOX2, Nanog and CD44) were detected in CBY1-overexpressed BCSCs by qRT-PCR (***P*<0.01). (D) qRT-PCR was used to assess the expression levels of CBY1, Nanog and SOX2 in breast cancer tissues (n=50).
