# Supplementary material for: A novel Lnc408 maintains breast cancer stem cell stemness by recruiting SP3 to suppress CBY1 transcription and increasing nuclear β-catenin levels
Source: Cell Death Dis. 2021 May 1;12(5):437. doi: 10.1038/s41419-021-03708-6 (PMC8088435; doi:10.1038/s41419-021-03708-6)
Supplement: Supplementary file 7 — Supplementary Table S1 [file 41419_2021_3708_MOESM7_ESM.docx]

| **Supplementary Table S1. The shRNA sequences used to silence the target gene** | |
| --- | --- |
| Gene name | Sequence (5’ to 3’) |
| lncRNA408-shRNA2# | GGAGGACCACACTTTCCAT |
| lncRNA408-shRNA3# | CCCAAAGAAAGGCATCTTT |
| SP3-shRNA1# | GGTTATACATGACACTGAA |
| SP3-shRNA2# | GATAGATAGTACAGGTATA |
| CBY1-shRNA1# | GCATTCTTTGGATCGATCA |
| CBY1-shRNA2# | GTGGACATCTTATTAGACA |
| Scramble-shRNA | TTCTCCGAACGTGTCACGT |
