# Supplementary material for: A novel Lnc408 maintains breast cancer stem cell stemness by recruiting SP3 to suppress CBY1 transcription and increasing nuclear β-catenin levels
Source: Cell Death Dis. 2021 May 1;12(5):437. doi: 10.1038/s41419-021-03708-6 (PMC8088435; doi:10.1038/s41419-021-03708-6)
Supplement: Supplementary file 8 — Supplementary Table S2 [file 41419_2021_3708_MOESM8_ESM.docx]

| **Supplementary Table S2. Primers used for qRT-PCR** | | |
| --- | --- | --- |
| Gene name | Forward 5’-3 | Reverse 5’-3’ |
| ENST00000422408 | TTTATCATGTGGCTACTAACGA | TGGGGACATTCAAACCATAGCA |
| ENST00000531827 | TGTCTGCCTGTTGTACCTTG | CCTCATTCTTTCACTTGCCAA |
| gnl_UG_Hs_S1269781 | TTAAATTTTCCTGGTGAACT | TGGCTATATTAACCATATGACA |
| ENST00000445540 | TTGACAGCCAGTATTCGCATC | TACCAGCACAGTCTTGTCC |
| ENST00000528792 | AGCCCAACTTTCTATAGCC | TAAATGAAACACTATTGCGGAGT |
| gnl_UG_Hs_S954271 | AATTTGAATATTTTGTTACCCTG | CAGCACCAAACCATACCAC |
| gnl_UG_Hs_S2961525 | AGCACATTTCCAGACAACGAA | CTCCAGGGGTACAATCCATC |
| ENST00000439336 | GGGCTCCAACAAATTAACAAGG | TACATTCCCAGGTGCTACACA |
| uc003euj | TCTTCTGAGACCGACAAGGC | GCGGGAGTCATCCTTAACCAA |
| ENST00000525233 | GCACCTACTGAAACCGGAA | TGCTGCTTGTATACTTTGTCC |
| CD44 | CTCTCGGACGGAGGCCGCTGACC | AGAAGGGCACGTGGTGATTCCCCG |
| SOX2 | GCACATGAACGGCTGGAGCAACG | TGCTGCGAGTAGGACATGCTGTA |
| Nanog | TTTGTGGGCCTGAAGAAAACT | AGGGCTGTCCTGAATAAGCAG |
| CBY1 | AAACGAGGACTCACTGTGCAA | CTTCGGACTGAACGTATTCCC |
| SP3 | CTTACTTGCCTCTGGAACACCT | ACCAAGAGGCACATTAGCAAC |
| KLF4 | GAACTGACCAGGCACTACCG | TTCTGGCAGTGTGGGTCATA |
| c-Myc | AAAGGCCCCCAAGGTAGTTA | GCACAAGAGTTCCGTAGCTG |
| β-Actin | TGACGTGGACATCCGCAAAG | CTGGAAGGTGGACAGCGAGG |
|  |  |  |
